# Are Weeds Hitchhiking a Ride on Your Car? A Systematic Review of Seed Dispersal on Cars

**DOI:** 10.1371/journal.pone.0080275

**Published:** 2013-11-12

**Authors:** Michael Ansong, Catherine Pickering

**Affiliations:** Environmental Futures Centre, Griffith University, Gold Coast, Queensland, Australia; BiK-F Biodiversity and Climate Research Center, Germany

## Abstract

When traveling in cars, we can unintentionally carry and disperse weed seed; but which species, and where are they a problem? To answer these questions, we systematically searched the scientific literature to identify all original research studies that assess seed transported by cars and listed the species with seed on/in cars. From the 13 studies that fit these criteria, we found 626 species from 75 families that have seed that can be dispersed by cars. Of these, 599 are listed as weeds in some part of the world, with 439 listed as invasive or naturalized alien species in one or more European countries, 248 are invasive/noxious weeds in North America, 370 are naturalized alien species in Australia, 167 are alien species in India, 77 are invasive species in China and 23 are declared weeds/invaders in South Africa. One hundred and one are classified as internationally important environmental weeds. Although most (487) were only recorded once, some species such as *Chenopodium album*, *Poa pratensis* and *Trifolium repens* were common among studies. Perennial graminoids seem to be favoured over annual graminoids while annual forbs are favoured over perennial forbs. Species characteristics including seed size and morphology and where the plants grew affected the probability that their seed was transported by cars. Seeds can be found in many different places on cars including under the chassis, front and rear bumpers, wheel wells and rims, front and back mudguards, wheel arches, tyres and on interior floor mats. With increasing numbers of cars and expanding road networks in many regions, these results highlight the importance of cars as a dispersal mechanism, and how it may favour invasions by some species over others. Strategies to reduce the risk of seed dispersal by cars include reducing seed on cars by mowing road verges and cleaning cars.

## Introduction

Weeds, which are often defined as undesirable plants growing in sites where they are not wanted, are a major threat to biodiversity globally [1-7]. They compete with native plants for resources such as nutrients, water and light, often resulting in reductions in the distributions of native plants and animals, as well as contributing to local and regional species extinctions [1-3,6,7]. Weeds also have economic impacts, including reduced productivity in many agricultural areas and increasing management costs for natural and agricultural systems [1-3]. In Australia, for example, in 2005 Paterson’s Curse (*Echium plantagineum*) was estimated to cost agriculture ~AU$30 million per year while Lippia (*Phyla canescens*) cost ~AU$38 million per year to the grazing industry [3,8]. The annual total cost for Japanese Knotweed (*Fallopia japonica*) to the British economy was estimated at over £165 million in 2010 [9].

Humans are important dispersal vectors for weed seed. We can deliberately introduce novel species into gardens, forests, pasture land and agricultural fields including, plants with weedy traits [3,5,10-13]. We can also accidentally carry and disperse weed seed on clothing, vehicles, domesticated animals, in soil and animal fodder [12,14-20]. Human-mediated dispersal is important when assessing plant invasions, as the seeds can unintentionally be dispersed over very long distances, including, between regions, countries and even continents [10,11,13,14,16,18-23]. The deliberate and unintentional introduction of species could contribute to the creation of a global weed flora, especially when different geographical locations increasingly receive similar weeds [13,16,24,25]. 

Individual studies, including those that collected seeds directly from the surface of vehicles or from car wash sludge [26-30], have found that vehicles can disperse the seed of a range of species, including many species of weeds. Seed from over 230 taxa, including 23 noxious weeds were identified from a range of vehicles in south-eastern Australia [29] while the annual seed rain from vehicles ranged from 635-1579 seeds/m^2^/year on the edges of roadside tunnels along a motorway in Berlin, Germany, many of which were not native to the region [31]. The diversity of seed dispersed by cars is very high in many studies, indicating that vehicles can be important mechanism for seed dispersal [26,28,32].

Seed unintentionally transported on/in cars can be dispersed over long distances and in many regions of the world [30,31,33-35]. The number of cars on the road and the size and extent of road networks is increasing rapidly in many regions. For example, there were ~17 million motor vehicles in Australia in 2012, of which 12.7 million were passenger cars up from 11.5 million in 2007 [36]. In the United States, the number of vehicles was 245 million in 2011 up from 230 million in 2001 [37], while in India there were 142 million vehicles up from 55 million vehicles over the same time period [38]. There were ~6.3 million cars registered in South Africa [39], 120 million cars in China [40] and 240 million cars in the European Union [41] in 2012.

While it is clear that unintentional human mediated seed dispersal via cars is an important mechanism for the dispersal of seeds including naturalized, invasive and noxious species [42,43,44], we still do not know: (1) how many and which species can be dispersed by this mechanism? (2) how many of them are weeds and where are they weeds? (3) are species with particular traits, growth forms and lifespans favoured by this dispersal mechanism? (4) how many seeds on average can be dispersed by a single car and were on a car are seed found? and (5) do driving conditions affect the amount of seed dispersed? To answer these questions, we systematically searched the academic literature to identify all original scientific research studies reporting species with seed dispersed by cars. We then compared the database of species with lists of regional and globally important weeds to assess the diversity of weeds that can be dispersed by cars. 

## Methods

### Systematic literature review

This systematic review conforms to the guidelines outlined by the Preferred Reporting Items for Systematic Reviews recommendations (PRISMA) [45] and follows the systematic quantitative literature review approach outlined in Pickering and Byrne [46]. We have used the PRISMA Statement to demonstrate how we conducted the review and reported the methods and results used in this review according the requirement of the PRISMA reporting criteria (Checklist S1).

To obtain a comprehensive list of species with seed that can be dispersed on/in passenger vehicles (cars), we conducted a systematic review of the academic literature to identify all original published research studies that: (1) assessed seed dispersed by passenger vehicles, and (2) either directly identified seed collected from vehicles or identified seedlings of seed collected from vehicles, and (3) included the scientific names of the species and (4) were published in English. 

To identify eligible studies, we conducted a systematic search of original research studies using the electronic databases: ScienceDirect, Web of Science, Scopus, Google Scholar and Google, from April 2012 to May 2013. A search string using a combination of the following keywords were used; man, human, tourists, visitors, automobile, motor vehicles, vehicles, cars, weeds, seeds, diaspores, spread, transport* and disper*. We used different combinations of search terms based on the requirements or limitations of each database. The search strategy for ScienceDirect, for example, was (man OR human OR tourists OR visitors OR motor vehicle* OR vehicle* cars OR automobile) AND (weeds OR seeds OR alien species OR invasive species OR non-native OR diaspores) AND (spread OR transport* OR disper*). The references cited by each potentially relevant study were reviewed to locate additional potential studies. 

**Figure 1:**
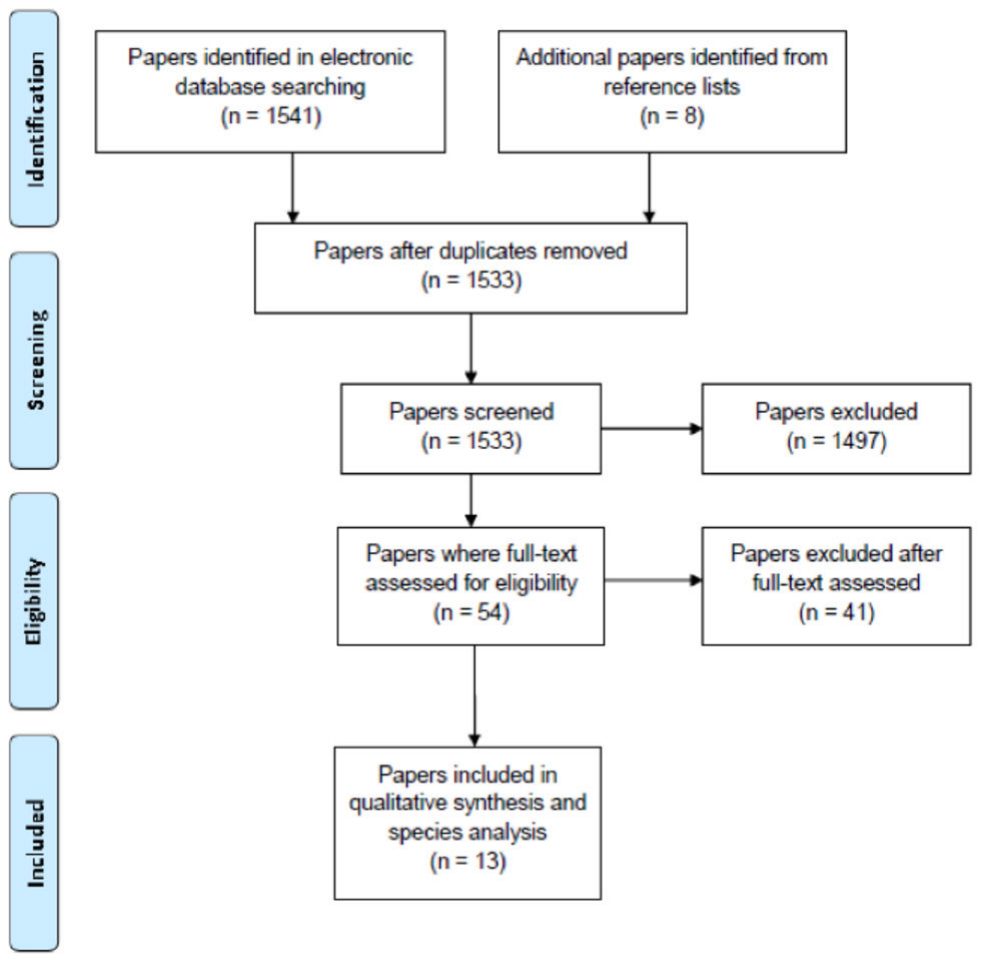
Flow chart using the PRISMA statement for the systematic review.

To ensure that we only included studies related to the topic, we first screened all the studies obtained after reading the titles and abstracts, and then excluded those that did not mention: 1) automobile or vehicles or humans as dispersal vectors; or 2) dispersal, or spread, or transportation, or 3) dispersal of plants, or weeds or alien species or invasive species. We then obtained the full text for all the original research study that passed this initial screening and reviewed them in detail. A second screening was then employed where a study was excluded if it did not generate empirical data or only reported on seed dispersed by vectors other than vehicles. Also, studies that reported specifically on specialized vehicles such as farm machinery/vehicles, earth moving vehicles, military vehicles, aircraft and boat/ships were excluded. Only studies that reported data about seed dispersed by general types of cars (sedans, two and four-wheel drive, utilities and wagons) were included. The number of studies excluded and those retained were recorded for each of the screening stages according to the PRISMA Statement (Figure 1) [45]. At the end, a total of 13 original research studies fitted all the criteria out of a total of 1,533 potentially relevant studies (Figure 1). For these 13 studies, information about the research in the studies was extracted including the name of author(s), publication year, geographical location of the study (continent and country), details of the sampling design, methods used to identify species (germinated seed and/or direct identification of seed) and the number of taxa listed in the study (Table 1). 

**Table 1:** Number of studies from 1959 to 2013 listing species that have seed collected from cars.

Reference	Country	Continent	Method of Id	Research design	Number of taxa in study	Species in database
Clifford [32]	Nigeria	Africa	GG	O	40	39
Wace [26]	Australia	Australia	GG	NE	259	94
Schmidt [27]	Germany	Europe	GG	O	124	34
Lonsdale and Lane [66]	Australia	Australia	DI	NE	88	26
Hodkinson and Thompson [30]	Britain	Europe	GG	NE	37	36
Zwaenepoel et al. [28]	Belgium	Europe	GG	NE	33	28
von der Lippe and Kowarik [31] and von der Lippe and Kowarik [47]	Germany	Europe	GG	NE	20	200
Moerkerk [29]	Australia	Australia	GG,DI	NE	20	222
Veldman and Putz [68]	Bolivia	S America	DI	NE	4	4
Rew and Fleming [64]	USA	N America	GG	NE	61	19
Nguyen [65]	Australia	Australia	GG	NE	146	135
Taylor et al. [33]	USA	N America	DI	ME	3	3
Auffret and Cousins [34]	Sweden	Europe	GG	NE	49	49

The table includes the continent where the research were performed, the methods used to identify the species, the research design, the number of taxa recorded in the study and the number of species from the study included in our database. Note: N America = North America, S America = South America. Method of identification: GG = seed germinated in glasshouse, DI = direct identification of seed. Experimental design: O = Opportunistic sampling (seeds were collected without any experimental design, but as part of other activities), NE = Natural experiment (involved some sort of experimental design but no, or limited, statistical testing), ME = Manipulative experiment (use of controls, randomization in the allocation of treatments, and statistical analyses of the results); Taxa in study = number of species, genera and morphotaxa listed in the study. Species extracted = total number of species identified.

Authors of three of the studies [29,31,47] kindly gave us more complete species lists than were included in the original studies. Two studies identified in the electronic searches [31,47] represented different aspects of the results from the same experiment and therefore were treated as one dataset (Table 1).

The studies are concentrated in a few regions, with five studies from Europe and four from Australia (Table 1). They had one of three research designs with 10 natural experiments (Table 1). Across the 13 studies, seeds were most often collected from mud or sludge with some of the studies reporting other methods such as brushing, trapping, vacuuming and/or hand removal from vehicle parts. Nine of the studies identified the species (456) based on seedlings germinated in glasshouses and/or laboratories (Table 1).

### List of species with seed dispersed on/in cars

**Figure 2:**
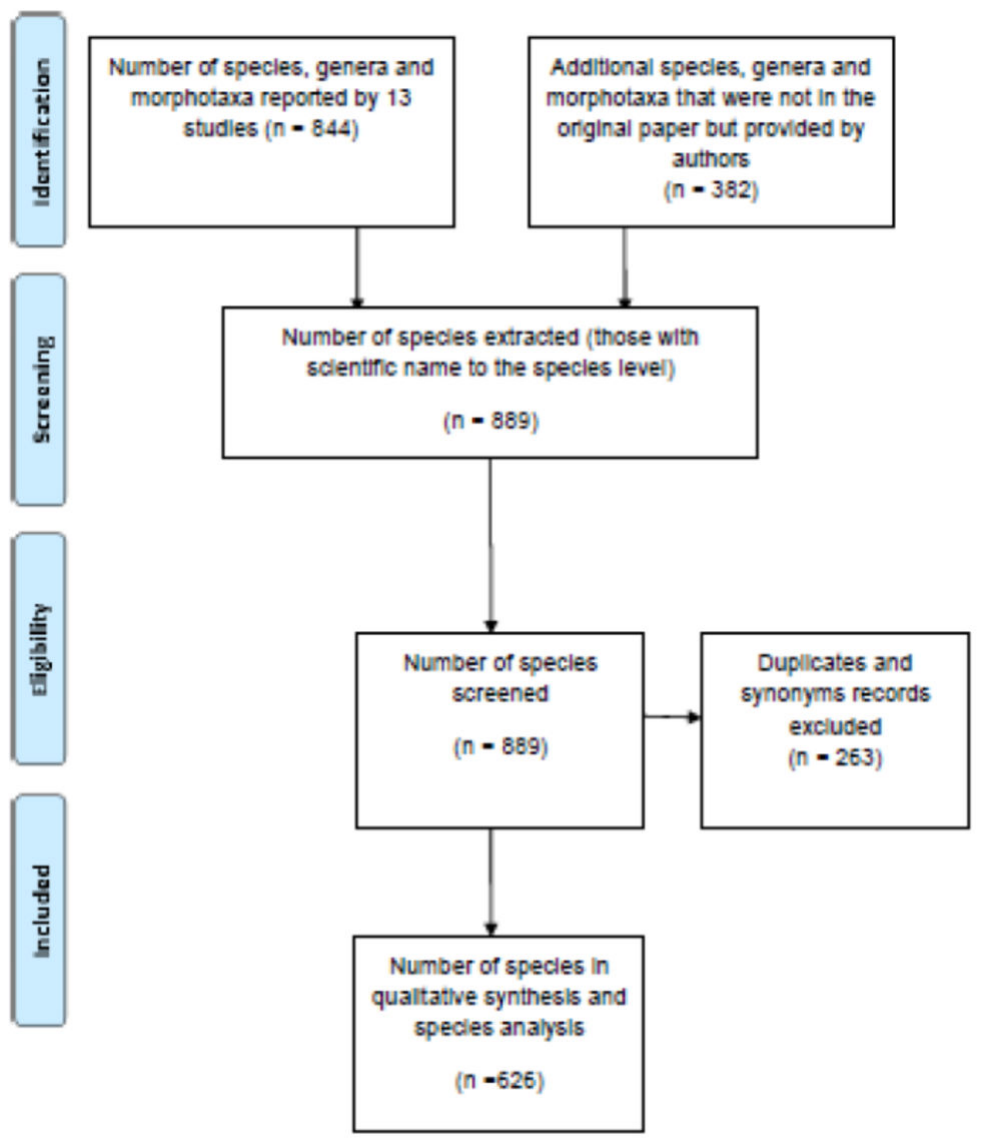
Flow chart using the PRISMA statement showing the steps involved in screening individual taxa. This was done to obtain a comprehensive list of species with seed collected from cars.

To obtain as complete a list as possible of species with seed dispersed by cars, we created a species database using results from the 13 studies. Due to variation in the methods used, duration and location of study and measurements taken among the studies, it was only possible to compare presence/absence data for species among the studies. To ensure consistency in the results, only data at the level of species was included, not genera, and only if the scientific name was included (e.g. not morphotaxa). The species database included information about all species listed in one or more of the studies, including its scientific name, common name, synonyms, family, growth form and life span. Species names were obtained from the original studies, while other information about those species was obtained from online databases and a weed compendium [43,48,49,50]. Plant nomenclature conforms to United States Department of Agriculture [49] and PlantNET [50]. Synonyms were checked and data consolidated by removing duplicates and retaining only names that conform to the nomenclature authority used (Figure 2). 

We used the Chi-square goodness of fit tests to compare the proportion of seeds of different growth forms and life spans collected on/in cars. The Chi-square test for independence was then used to determine if growth form of the species with seed dispersed by cars were dependent on their life spans. The categories, annual, biennial and those species that are both annual and biennial species were combined and referred to here as ‘annual-biennial’ while those with mixed or intermediate life spans such as biennial/perennial and annual/perennial were combined to form a category called ‘mixed’.

### Weed status of species

To determine if a species was considered a weed somewhere in the world, data from the ‘*Global Compendium of Weeds*’ was used [48]. For international environmental weeds, that is, weeds that invade natural vegetation and adversely affect biodiversity, we used a list of the top 400 international environmental weeds [43]. Our database was also compared with the list of the 100 world's worst invasive alien species [51]. 

To assess how many species of weeds in Europe have seed that could be dispersed by cars, we compared our database with the ‘European Alien Species Database’ from the Delivering Alien Invasive Species Inventories for Europe (DAISIE) [52]. This database includes 5,970 species classified as alien invasive/naturalized plants in one or more of the 27 European Union member countries [53] including the 150 most widespread alien species in Europe [54]. We also assessed the invasive status of the species according to information on alien and invasive plant species in north and central Europe provided by The European Network on Invasive Alien Species (NOBANIS) [55]. The 52 species on the European and Mediterranean Plant Protection Organization (EPPO) invasive plant species list [56] was also used. To see if car dispersal is common in the weed flora of a specific country in Europe we used the catalogue of invasive species for the Czech Republic, which comprises 1,454 alien taxa of which 61 are considered invasive [57]. Weed status in countries were the studies were carried was also determined.

Data on the weed status in Australia of the species in our database were obtained from the 2,739 species of weeds that are listed as naturalised in Australia [11]. We also compared our database with the 429 plant species declared ‘Noxious Weeds’ by one or more state or territory government [44] and the 32 weeds that are considered of ‘National Significance’ as determined by the Australian Government [58]. To assess how many weed species in North America could be dispersed by cars, we used the 1,596 species that have been documented as invasive or noxious weeds in North America [59] and the 112 plant species declared noxious weeds federally in the United States [60]. As a single comprehensive list of invasive species for Asia and Africa was difficult to obtain, so we focused on weed lists for India, China and South Africa, which have large human populations, many cars and expanding road networks. For India, we used a list of 1,599 species classified as alien plant species, of which 225 species are declared invasive [61], while for China we used a list of 265 invasive plant species [62]. For South Africa, we used the list of 198 species of weeds/invasive plants listed in Regulation 15 of ‘*The Conservation of Agricultural Resources Act*’, No. 43 of 1983 of South Africa [63]. 

## Results

### Which species have seed that can be dispersed by cars?

**Figure 3:**
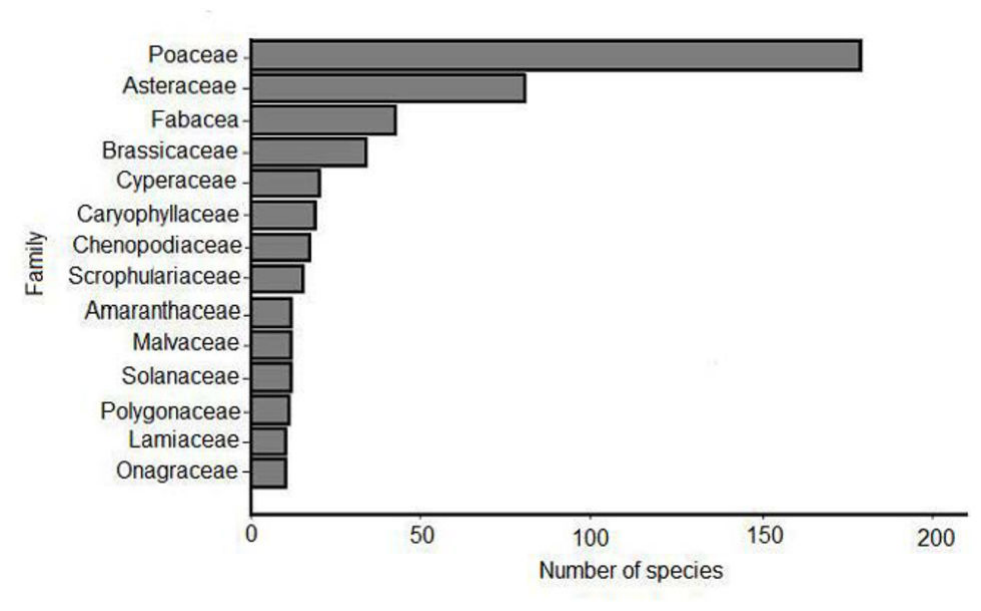
The number of species from the most common plant families with seed collected from cars.

Seeds of 626 species representing 75 families have been collected from cars (Table 2, Figure 2); with an average of 48 species reported per study (range 2-222). The families with the greatest diversity of species were the Poaceae (grasses) with 178 species (28%), the Asteraceae (daises, 80 species, 13%) and the Fabaceae (peas, 42 species, 7%) (Figure 3).

**Table 2:** Summary of the number of species with seed collected from cars, weed status globally, in Europe, Australia, North America, China, India and South Africa, their life forms and growth forms.

Category	Number of species
Total species	626
Weed status globally	
Weed	599
Environmental weed	101
100 world's worst invasive	4
Weed status in Europe	
Declared weed species	439
150 most widely naturalized species	55
Invasive in Europe	63
EPPO list of invasive species	3
Declared weed in Czech Republic	191
Invasive in Czech Republic	18
Weed status in North America	
Listed as invasive or on a Noxious weed in North America	248
Declared noxious in the United States	14
Weed status in Australia	
Naturalized	370
Declared invasive	21
Weeds of National significance	9
Declared noxious	64
Weed status in India	
Declared weed	167
Declared invasive	56
Declared invasive in China	77
Declared invasive in South Africa	23
Growth form	
Graminoids	202
Forbs	360
Tree	31
Shrubs	33
Life span	
Annual	238
Annual/biennial	36
Annual/perennial	50
Biennial	7
Biennial/perennial	6
Perennial	289

Note: Weed status was according to Randall [48]. Environmental weed was according to Weber (2003). among 100 world's worst invasive according to Lowe et al. [51] Weed in some part of Europe [52]; Invasive in Europe according to The European Network on Invasive Alien Species [55], In vasive according to European and Mediterranean Plant Protection Organization (EPPO) invasive plant species list [56], among 150 most widespread alien species in Europe [54] and status in Czech Republic [57]. Invasive/noxious in North America was according to Invasive.org [59]. Naturalize in Australia [11]. Noxious or a weed of national significance was according to The Australian Weeds Committee [44,58]. Weed status in India [61], in China [62] and in South Africa [63].

When seeds of species on cars were compared to plants growing in the region, many of the species with seed on cars are also found growing on road verges and/or in the local area [26-28,30,32,34]. However, the frequency and/or abundance of seed on cars compared to  plants in the local area is often quite low, indicating that car floras are a selective mechanisms, favouring the dispersal of seed of a subset of species found on road verge flora [27,28,30,32].

### How many of these species are weeds and where are they weeds?

Nearly every species with seed collected from cars has been cited as a weed in some part of the world (599, 96%) [48], with over one-sixth (101 species) are classified as international environmental weeds [43] and some are also among the 100 world's worst invasive species [51] (Table 2). 

At least 439 of the 5,970 alien invasive/naturalized species recorded in one or more European countries [52] have seed that can be dispersed by cars (Table 2). These include one-third of the 150 most widespread alien species in Europe [54] and 13% of the alien flora of the Czech Republic (Table 2). Many are considered particularly threatening including *Buddleja davidii*, *Heracleum mantegazzianum*, *Paspalum distichum* and *Solidago canadensis* which are considered highly invasive and threaten plant health and/or biodiversity (Table 2) [56].

Almost half (206) of the 439 invasive/naturalized species in Europe had seed collected in one or more of the five studies conducted in Europe. Across the three studies in Germany [27,31,47], there was a total of 213 species with seed from cars, of which 65 (31%) are invasive/naturalised in Germany. Of the 49 species with seed on cars from the one study in Sweden [34], 29 (59%) are naturalized alien species in Sweden, two of which, *Stellaria media* and *Urtica dioica* are declared invasive in Sweden [55]. For the one British study [30], 9 (25%) of the 36 species found with seed on cars was invasive/naturalized in Britain, while for the one study from Belgium [28], three (11%) of the 28 species were invasive/naturalized in Belgium [52]. These results demonstrate that a wide diversity of weed species in Europe is dispersed by cars in Europe.

Many non-native plants in Australia have seed that can be dispersed by cars. This includes, 14% of the 2,739 naturalised plants in Australia, ~15% of the 249 declared noxious weeds and 28% of the 32 species of weeds considered of national significance in Australia (Table 2). Across the four studies conducted in Australian, 256 species were recorded that are not native to Australia, including the international invasive weeds *Andropogon gayanus*, *Chrysanthemoides monilifera, Genista linifolia, Nassella neesiana, Nassella trichotoma, Rubus fruticosus* and *Ulex europaeus* (Table 2) (Species List S1). 

Although only two of the studies were from North America [33,64]; 16% of the 1,596 species listed as invasive/noxious in North America [59] can be dispersed by cars. This included 15% of the nationally listed noxious weeds of the United States [60]. The two American studies, however, recorded only seven species that are considered invasive/noxious weeds in North America, but this included the international environmental weeds *Salsola kali*, *Poa pratensis* and *Verbascum thapsus* [33,43,64].

Although no original research studies from India, China and South Africa were found in the systematic search of the literature, the database included many species considered to be weeds in these countries. One tenth of the 1,599 alien species in India, including a quarter of the 225 invasive species have seed that has been collected from cars (Table 2). Almost a third of the 265 invasive species in China [62] have seed that has been collected from cars. About 12% of the 198 plant species declared as weeds or invaders under Regulation 15 of The Conservation of Agricultural Resources Act, No. 43 of 1983, of South Africa [63] can also be dispersed by cars.

### Which plant and seed traits favour dispersal by cars?

Among the 625 species with seed dispersed by cars, most were forbs (58%) or graminoids (32%) with few shrub or tree species (χ^2^ = 210.3, d.f. = 2, P < 0.001) (Table 2). There were nearly equal numbers of perennial species as annual/biennial but few mixed species with seed (χ^2^ = 167.7, d.f. = 2, P < 0.001) (Table 2). When life span and growth form were examined together, there were significant differences; with 19% more perennial graminoids than annual graminoids, 26% more annual forbs than perennial forbs dispersed by cars (χ^2^ = 105.2, d.f. = 4, P < 0.001).

Although there was considerable variation in the species recorded among the studies, with 487 species recorded once (Species List S1), there were some common species (Table 3 and Table 4). For example, there were 16 species recorded in five or more studies, which had some traits in common (Table 3 and Table 4). All 16 species, for instance, are considered widespread species that benefit from disturbance (Table 4), have very high seed output, release seed around ≤ 1 m from the ground, and have small/light seed (mostly ≤ 1 mg) (Table 3). Most can also reproduce vegetatively (Table 4). They were all forbs (nine) or graminoids (seven), with no shrubs or trees, and 50% each annuals and perennials (Table 3). Nearly all of them grow in open habitats and have a low tolerance for shade (Table 4). 

**Table 3:** Biological traits of the most common species recorded in five or more of the 13 studies on seed from cars.

Species	Family	Growth form	Life form	Shoot growth form	Mean release height (m)	Presence of seed bank	Morphology of seed	Seed per ramet/tussock/individual plant	Mean seed mass (mg)
*Sonchus asper*	Aster	Forb	Annual	Stem erect	0.5-1.0	Transient	Pappus	≤100,000	0.1-0.4
*Sonchus oleraceus*	Aster	Forb	Annual	Stem erect	0.5-1.0	Short term persistent	Pappus	≤10,000	0.1-0.4
*Taraxacum officinale*	Aster	Forb	Perennial	Ascending to prostrate	0.5-1.0	Transient	Pappus	≤10,000	0.5-1.0
*Stellaria media*	Caryo	Forb	Annual	Ascending to prostrate	0.1-0.4	Shoot growth form		≤100,000	0.1-0.4
*Sagina procumbens*	Caryo	Forb	Perennial	Ascending to prostrate	0.1-0.4	Short term persistent		≤10,000	<0.1
*Chenopodium album*	Cheno	Forb	Annual	Stem erect	0.5-1.0	Short term persistent		≤100,000	0.5-1.0
*Trifolium repens*	Fab	Forb	Perennial	Prostrate	0.1-0.4	Transient		≤1000	0.5-1.0
*Juncus bufonius*	Junc	Graminoid	Annual	Stem erect	0.1-0.4	Short term persistent	Sticky	≤100,000	<0.1
*Plantago major*	Planta	Forb	Perennial	Stem erect	0.1-0.4	Shoot growth form	Sticky	≤100,000	0.1-0.4
*Poa pratensis*	Poac	Graminoid	Perennial	Ascending to prostrate	0.5-1.0	Transient		≤1000	0.1-0.4
*Poa annua*	Poac	Graminoid	Annual	Prostrate	0.1-0.4	Short term persistent	Hairs	≤100,000	0.1-0.4
*Dactylis glomerata*	Poac	Graminoid	Perennial	Stem erect	0.5-1.0	Transient	Hairs	≤1000	0.5-1.0
*Echinochloa crus-galli*	Poac	Graminoid	Annual	Ascending to prostrate	0.5-1.0	Short term persistent	Awns	≤100,000	2.0-2.5
*Lolium perenne*	Poac	Graminoid	Perennial	Stem erect	0.5-1.0	Transient		≤100,000	1.5-2.0

Note: **Family**: Aster- Asteraceae; Caryo- Caryophyllaceae; Cheno- Chenopodiaceae; Fab- Fabaceae; Junc- Juncaceae; Planta- Plantaginaceae; Poac- Poaceae; Polug- Polygonaceae. The details about the number of seed, presence of seed bank, seed mass, release height, and shoot growth form were obtained from the list of 400 international environmental weeds [43], PlantNET [50] and the United States Department of Agriculture [49].

**Table 4:** The extent of the distribution, adaptions and weed status in Australia, North America and Europe of the most common species recorded in five or more of the 13 studies on seed from cars.

Species	Shade tolerance	Widely distributed	Benefits from disturbance	Vegetative organs	Weed somewhere	Naturalize in Australia	Invasive in North America	Weed in some part of Europe	Number of studies
*Sonchus asper*	Low	Yes	Yes	Yes	Yes	Yes	Yes	Yes	6
*Sonchus oleraceus*	Low	Yes	Yes	Yes	Yes	Yes	Yes	Yes	5
*Taraxacum officinale*	Low	Yes	Yes		Yes	Yes	Yes	Yes	5
*Stellaria media*	Low	Yes	Yes	Yes	Yes	Yes		Yes	6
*Sagina procumbens*	Low	Yes	Yes	Yes	Yes	Yes		Yes	5
*Chenopodium album*	Low	Yes	Yes		Yes	Yes	Yes	Yes	7
*Trifolium repens*	Low	Yes	Yes	Yes	Yes	Yes	Yes	Yes	7
*Juncus bufonius*	Low	Yes	Yes	Yes	Yes	Yes		Yes	6
*Plantago major*	Low	Yes	Yes		Yes	Yes	Yes	Yes	6
*Poa pratensis*	Low	Yes	Yes	Yes	Yes	Yes	Yes	Yes	7
*Poa annua*	Low	Yes	Yes		Yes	Yes	Yes	Yes	6
*Dactylis glomerata*	High	Yes	Yes	Yes	Yes	Yes	Yes	Yes	5
*Echinochloa crus-galli*	Low	Yes	Yes		Yes	Yes	Yes	Yes	5
*Lolium perenne*	High	Yes	Yes	Yes	Yes	Yes	Yes	Yes	5

Note: Invasive in North America if in the invasive species list or noxious weed law in North America [59]; Weed in some part of Europe if in The European Alien Species Database of the Delivering Alien Invasive Species Inventories for Europe (DAISIE) database [52 ]; Naturalized in Australia if in the list of introduced flora of Australia [11]; Weed according to Randall [48]. Shade tolerance and extent of distribution was obtained from the list of 400 international environmental weeds [43], PlantNET [50] and the United States Department of Agriculture [49].

Some similar results were reported in individual studies. Species that have seed transported over long distances by cars often produced a lot of seeds [34] that are mostly small in size and lighter in weight [27,28,30,32], and have a persistent seed bank [28,30,34]. Seeds dispersed on cars have also been found to be from low-growing (releasing) plants [34] and from species with shorter seeds [28]. 

### How much seed and how far can they be dispersed by cars?

Collectively cars can transport a large number of seeds with estimates of 67,500 ± 7,300 seedlings per ton of dry sludge from car washes in Australia [65], 6,252 seedlings germinating in glasshouses from seed traps located inside long motorway tunnels in Germany [31] and 3,926 seedlings germinating from mud collected from cars driven in Germany [27]. Estimates of the number of seed per car, however, tend to be low with estimates of 3-6 seeds per tourist car in a National Park in northern Australia [66], 1-2 seeds per vehicle in University of the Sheffield car parks in England [30] and three seeds per vehicle in a car park at the University of Ghent in Belgium [28].

When the results of the studies that estimated number of seed per car [28,30,66] are combined we estimate an average of only 2-4 seeds could be carried by each car. Multiplying these numbers by the current number of cars in a country or region it is possible to estimate the average number of seeds that could be transported by this vector. For example, cars could be moving around ~490-980 million seeds in the United States, 480-960 million seeds within the European Union, 248-496 million seeds in India, 240-480 million seeds in China, 25.4-50.8 million seeds in Australia, and 12.6-25.2 million seeds in South Africa [36,37,38,39,40,41]. 

Only one study [33] has quantified the actual distance seed can be transported by a car. This was done using a manipulative experimental design to assess the dispersal potential of Bread wheat (*Triticum aestivum*), Rapeseed (*Brassica napus*) and Wild rye (*Elymus trachycaulus*) which found that some seeds were transported over 250 km. Some other studies have estimated the distance seed could be dispersed by cars, with Hodkinson and Thompson [30] indicating that while seed may be dispersed over hundreds of kilometres they were most likely to be dispersed between 3-40 km. Other studies indicated shorter distances, with von der Lippe and Kowarik [31] estimating that seeds of at least 32 species could be dispersed over 250 m while Veldman and Putz [68] estimated that seed had travelled at least 500 m on cars.

### Where can seed collect on/in cars?

Seeds have been collected from different parts of cars including under the chassis, front and rear bumpers, wheel wells and rims, front and back mudguards, wheel arches, tyres and the interior floor mats. Considerable variation in the amount/diversity of seed among different parts of a car has been recorded [29,33,67]. In an Australia study, more seeds were found attached to the underside of the cars, followed by back mudguards, front mudguards and cabins than on engines, radiators, tyres and rims [67]. In terms of the diversity of seed, cabins and engine bays have been found to contain a greater diversity of viable seed than other parts of cars in another study in Australia [29]. In a study determining whether long distance dispersal by vehicles contributes to grass invasion in Bolivian forests [68], most seeds were found on the tires and wheel wells and on interior floor mats. This was related to the amount of mud attached to, or deposited in, the car and how often that part of the car was exposed to air movement and/or wet conditions [29,33,67]. Some studies indicate that seeds are dispersed over shorter distances when attached to more exposed parts of a car such as wheel wells, undersides and the front and rear bumpers, especially in wet conditions [28,33,64,67].

### Do driving conditions influence type and amount of seeds dispersed?

Based on the results of the studies, the potential for seed to be dispersed by cars is affected by several factors including where cars are driven [33,64]. Variation in the amount and type of seed among cars used for different purposes and at different times was observed in some of the studies [64,66]. Cars traveling on unpaved/untarred roads and off-road for instance, often collected more seed in mud/soil than those driven on paved roads, particularly if conditions are wet [32,33,64]. As a result, cars used by more adventurous tourists/drivers were reported to collect/disperse more seed because they were mostly driven on unpaved roads and/or off-road with lots of mud that contains seeds [33,64,66]. 

Climatic conditions can affect seed dispersal by cars. For example, more seeds were found on cars driven under dry conditions either on paved and unpaved roads than driven under wet conditions, irrespective of the location of seed on the car [28,33,64]. Although in wetter conditions, mud on the road increased the risk of seeds attaching to cars, particularly on unpaved roads, it was thought to also increase the potential for mud and seed to be washed off cars [33], while dry conditions facilitated longer distance dispersal [28,33,64]. 

According to some of the studies [28,30,32,65], seasonality can also affect seed dispersal by cars. More seeds were found on cars when plants are seeding, including more seed on cars autumn in Australia [67] or in the dry season in Nigeria [32] than at other times of year when fewer species were seeding. 

## Discussion

Cars can unintentionally transport seeds from a diversity of plants, including a wide range of weeds. So far, nearly all (96%) the species with seed collected from cars are weeds in some part of the world [48]. They also include globally invasive species including, over a quarter of 400 international environmental weeds [43], and some of the world's worst invasive species [51]. Although far from exhaustive, the results of the current studies highlight the importance of this type of unintentional human mediated seed dispersal. 

The spread of weed seed by cars is a global-scale problem. As estimated in this review, at least 14% of weed species naturalized in Australia, 10% of alien species in India, 32% of invasive species in China, 15% of the noxious weeds of the United States and 13% of invasive species in South Africa could be transported by this mechanism. Many of the species recorded in the individual studies are also either established or invasive species in the countries were the studies were undertaken. Given the increasing numbers of cars, globally and in many of these regions, there is increasing risk of weed seed being dispersed in previously remote areas [14,19,20]. 

Many weed seeds that are dispersed by cars remain viable. For instance, in this review, 456 species were recorded germinating from seeds collected from cars. The importance of dispersal of viable weed seed is well recognised, including the threat they pose to the survival, abundance and distribution of many native species [13,16,19,24,25]. This is particularly important as even moderately resistant communities can be invaded when propagule pressure is high enough [4]. 

Life history and the growth form of species seem to be important for this dispersal mechanism. Cars appear to be far more likely to carry the seed of forb and graminoid species, rather than shrubs or trees. Perennial graminoids seem to be favoured over annual graminoids while annual forbs are favoured over perennial forbs. Several factors including seed size, seed morphology and where the plants grow may contribute to these differences. It is possible that the predominance of seed from annual forbs on cars reflects their smaller seed sizes, large numbers of seed and dominance in highly disturbed sites such as road verges where seed may more easily become attached to cars. Season, environment/weather conditions, where the car is driven and the parts of cars exposed to the elements also affect this type of seed dispersal [28,31,33,64,68]. 

Despite over 50 years of published research on seed dispersal by cars and increasing concern about long distance seed dispersal mechanisms, there are still surprisingly few studies on this topic in the academic literature. Of even more concern are the geographical gaps in the research, with no studies found from Asia, Central America and New Zealand, and only one studies each from Africa [32] and South America [68]. Further research, particularly in areas currently poorly represented in the literature such as Asia and Africa, are likely to add many new species to those with seeds currently recorded from cars. Studies that compare species collected on cars with regional/local species pools [26-28,30,32,34] should be conducted to test the role of different seed traits on this dispersal mechanism. 

With the high diversity of weed seed found on cars it is important to minimise the risk of this type of seed spread. Relatively straight forward methods to reduce the risk of this type of seed dispersal include mowing road verges before plants are seeding to prevent the seed of weeds attaching to cars [18,22,64]. Cleaning cars regular, particularly before and after driving on unpaved roads and/or in muddy conditions will help reduce the change of transporting mud that may carry weed seed. Similarly cleaning vehicles and other machinery prior to entry and use within areas of high conservation values such as national parks should be promoted. The duration and type of car washes should also be considered, as some car washes already promoted to reduce the spread of weed seed are not completely effective at removing soil/mud [33,64]. Effective education including for people using conservation areas, about the risk of spreading weed seed may help reduce the potential risks of weed dispersal by this vector.

## Conclusion

In this review we have demonstrated that seed from a wide diversity of weeds can ‘hitchhike’ a ride on cars. Among the traits common among species collected in these studies are the ability to produce large amounts of seed and have persistent seed banks; traits which have also been suggested to increase species invasiveness [6,7]. Despite the geographical limitations of current research, some general patterns are likely to be broadly applicable: the size and morphology of the seed, the plants life history, and where the plant grows. 

This review did not consider the number of seed per species, or if species went on to establish viable populations once dispersed. The high number of international environmental weed species collected, however, highlights the potential threat posed by this dispersal mechanism for biodiversity in all regions. The precautionary principle indicates that the risk of introducing weed seeds should be minimized, since propagule pressure is an important predictor of the plant invasions. We recommend more research on this and other types of unintentional human mediated seed dispersal including in regions such as Africa and Asia where the human population and the numbers of cars are increasing.

## Supporting Information

Checklist S1 **Checklist of items to include when reporting a systematic review or meta-analysis.**
(DOC)

Species List S1 **The families, life forms and growth forms of 626 species with seed collected from cars in the 13 papers reviewed.** The list also includes their weed status globally, in Europe, Australia, North America, China, India and South Africa.
(XLSX)
